# Note on the Germicidal Power of Flavine

**Published:** 1917-11

**Authors:** 


***Note on the Germicidal Power of Flavine.*** By R. TANNER HEWLET, Professor of Bacteriology in the University of London (Bacteriological Department of King's College). Abstracted from the *Lancet*, Sept. 29, 1917.

H. questions the conclusions of Browning and his co-workers[1] as

1. Browning, Gulbransen, Kennaway, and Thornton, *Brit. Med. Jour.*, 1917, vol. I, p. 73 (also ib., vol. II, p. 70). (See the preceding abstract for the substance of the article here criticized.)

to the great potency of flavine as a germicide, especially in serum. B. claims that flavine is germicidal in 24-48 hours at 37° C. in a strength of 1 : 20,000 in peptone water, and of 1 : 200,000 in serum.

H. objects that B. in tests made with staphylococcus and B. coli used a poor culture medium (peptone water). H. believes that the "20 or more colonies" obtained from a loopful of a 1 : 20,000 dilution of the 24-hour culture, were an insufficient number of organisms on which to make satisfactory tests, because, "there is an enormous destruction of organisms at an early stage, but a small number survives for some time. If only a few organisms are subjected to experiment, this factor may be entirely masked".

H. further objects to B.'s assumtion that effects produced upon serum will be similar to those produced upon pus, because serum is much the poorer culture medium for many organisms. H. cites B.'s own admission that a strength of 1 : 2000 flavine was needed to sterilize a streptococcus + staphyloccocus pus, as against his assertion that a strength of 1 : 200,000 is sufficient for serum cultures.

Even if flavine were stronger, H. believes it is too slow in operation (a 24-hour contact is needed) to replace less potent but more rapid germicides.

As to B.'s contention that flavine possesses low toxicity for leucocytes, H. believes that 20 minutes allowed in B.'s test for phagocytosis was insufficient, especially since 24 hours are required for toxic action on bacteria.

H. has himself made 6 tests with the following results :

1. B. perfringens (B. aerogenes capsulatus) group in milk (dust, recently boiled milk and flavine, in eight tubes, heated to 80° C. for 3 days). Conclusion : inhibitory power of flavine for this group is between 1 : 4000 and 1 : 10,000.

2. Staphylococcus in broth (flavine added to 5 cc. broth and .5 cc. 24-hour broth culture and incubated at 37° C. .1 cc. was then subcultured into broth after 9 hours and 24 hours). Conclusion : germicidal power of flavine on staphylococcus is between 1 : 5000 and 1 : 10,000 in 9 hours; and between 1 : 10,000 and 1 : 100,000 in 24 hours.

3. Staphylococcus in saline. Same results as in 2.

4. Staphylococcus in saline but with a much larger number of staphylococci (4000 million per cc.). Conclusions : the germicidal power of flavine is between 1 : 2000 and 1 : 4000 (much less than in 1 and 2).

5. Staphylococcus in human serum. Strengths of flavine 1 : 100,000 1 : 200,000 ; and 1 : 400,000 resulted in none killed. Serum to which,

.05 cc. broth culture of staph. was added, was then tested with flavine in strengths varying from 1 : 10,000 to 1 : 100,000). Conclusions : the germicidal power of flavine in serum is between 1 : 15,000 and 1 : 20,000 (B. had found it to be 1 : 200,000).

6. Staphylococcus in pus. (1 cc. staphylococcic pus and 1 cc. flavine solution incubated. Loopfuls sown in broth and on to agar). Conclusions : In agar subcultures made after 24 hours' incubation, 1 : 200 flavine did not completely kill. In broth subcultures the germicidal strength was between 1 : 200 and 1 : 1000.

The author attributes the poorer results thus obtained to the fact that he used much larger numbers of organisms than did B. He notes that results in pus were especially bad — no better if not worse than results from many disinfectants the action of which is much more rapid. He also calls attention to the fact that Fleming (*Lancet*, Sept. 1,1917, p. 341) reaches conclusions similar to his own.

